# *Trypanosoma cruzi* Disrupts Thymic Homeostasis by Altering Intrathymic and Systemic Stress-Related Endocrine Circuitries

**DOI:** 10.1371/journal.pntd.0002470

**Published:** 2013-11-14

**Authors:** Ailin Lepletier, Vinicius Frias de Carvalho, Patricia Machado Rodrigues e Silva, Silvina Villar, Ana Rosa Pérez, Wilson Savino, Alexandre Morrot

**Affiliations:** 1 Laboratory of Thymus Research, Oswaldo Cruz Institute, Oswaldo Cruz Foundation, Rio de Janeiro, Brazil; 2 Laboratory of Inflammation, Oswaldo Cruz Institute, Oswaldo Cruz Foundation, Rio de Janeiro, Brazil; 3 Institute of Immunology, Faculty of Medical Sciences, National University of Rosario and CONICET, Rosario, Argentina; 4 Laboratory of Immunobiology, Paulo de Goes Institute of Microbiology, Federal University of Rio de Janeiro, Rio de Janeiro, Brazil; Federal University of São Paulo, Brazil

## Abstract

We have previously shown that experimental infection caused by *Trypanosoma cruzi* is associated with changes in the hypothalamus-pituitary-adrenal axis. Increased glucocorticoid (GC) levels are believed to be protective against the effects of acute stress during infection but result in depletion of CD4^+^CD8^+^ thymocytes by apoptosis, driving to thymic atrophy. However, very few data are available concerning prolactin (PRL), another stress-related hormone, which seems to be decreased during *T. cruzi* infection. Considering the immunomodulatory role of PRL upon the effects caused by GC, we investigated if intrathymic cross-talk between GC and PRL receptors (GR and PRLR, respectively) might influence *T. cruzi*-induced thymic atrophy. Using an acute experimental model, we observed changes in GR/PRLR cross-activation related with the survival of CD4^+^CD8^+^ thymocytes during infection. These alterations were closely related with systemic changes, characterized by a stress hormone imbalance, with progressive GC augmentation simultaneously to PRL reduction. The intrathymic hormone circuitry exhibited an inverse modulation that seemed to counteract the GC-related systemic deleterious effects. During infection, adrenalectomy protected the thymus from the increase in apoptosis ratio without changing PRL levels, whereas an additional inhibition of circulating PRL accelerated the thymic atrophy and led to an increase in corticosterone systemic levels. These results demonstrate that the PRL impairment during infection is not caused by the increase of corticosterone levels, but the opposite seems to occur. Accordingly, metoclopramide (MET)-induced enhancement of PRL secretion protected thymic atrophy in acutely infected animals as well as the abnormal export of immature and potentially autoreactive CD4^+^CD8^+^ thymocytes to the periphery. In conclusion, our findings clearly show that *Trypanosoma cruzi* subverts mouse thymus homeostasis by altering intrathymic and systemic stress-related endocrine circuitries with major consequences upon the normal process of intrathymic T cell development.

## Author Summary

It is currently estimated that 90 million people in America are exposed to *T. cruzi* infection, the causative agent of Chagas disease. Despite the mortality and morbidity, this infection is yet considered a neglected disease, due to the lack of effective, safe, and affordable pharmaceuticals for controlling it. *T. cruzi* leads to immunosuppression of the T cell compartment and to chronic cardiac inflammation, which seems to be associated with the disruption of thymic homeostasis. Thymus atrophy, characteristic of acute infection, is mainly associated with the loss of immature CD4^+^CD8^+^ thymocytes, which in turn is associated with increased corticosterone systemic levels, together with their premature export to the periphery as potential autorreactive cells. Although being deleterious to the thymus, GCs are protective during this infection, for avoiding an exacerbated pro-inflammatory response. Here we demonstrate that the increase of GCs in plasma is related to the impairment of PRL systemic levels. The intrathymic hormonal circuitry is also altered during infection and an imbalance of the cross-talk involving GR and PRL is related with CD4^+^CD8^+^ depletion. The partial restoration of PRL levels prevented thymus atrophy of infected mice, thus partially reverting the *T. cruzi*-induced subversion of the organ, ultimately reestablishing thymus homeostasis.

## Introduction

The thymus is a primary lymphoid organ in which bone marrow-derived T cells differentiate into mature T lymphocytes. Thymus homeostasis is disrupted in several infectious diseases, including acute infection by *Trypanosoma cruzi*, the causative agent of Chagas disease [1], [2]. Accordingly, *T. cruzi* acutely-infected mice exhibit an intense thymic atrophy mainly characterized by a massive loss of cortical CD4^+^CD8^+^ thymocytes and an aberrant output of immature T cells, which have likely bypassed the negative selection process and may be involved with the generation of T cell autoimmune events seen in both murine and human Chagas disease [3]–[5].

It is well known that both prolactin (PRL) and glucocorticoid (GC) receptors (PRLR and GR, respectively) are constitutively expressed in thymocytes [6], [7], and their activation is related to the control of many aspects of thymus physiology. Besides participating in intrathymic T cell selection events, GCs inhibit thymocyte proliferation and induce apoptosis of these cells, acting via a specific receptor, inducing cell death through a caspase activation pathway [8]–[11]. On the other hand, PRL stimulates intrathymic T-cell proliferation and inhibits GC-induced thymocyte apoptosis [12], [13]. A further important issue concerning thymic homeostasis and stress hormones is the fact that, in addition to the endocrine function of GCs and PRL upon thymocytes and thymic microenvironmental cells, both hormones are produced intrathymically, and likely act locally through autocrine and/or paracrine loops [7], [14], [15].

The precise mechanisms triggering thymic involution following *T. cruzi* infection are not completely elucidated but seem to be, at least partially related to the rise of GCs systemic levels, a well-known effect comprised within the complex stress response to acute infections [1], [16]. Interestingly, in addition to increasing systemic GCs levels, infection affects PRL contents, another stress hormone that seems to counteract certain GC effects in the immune system [17], [18]. During *T. cruzi* infection, although the increased circulating levels of GCs can be protective by impeding an exacerbated production of pro-inflammatory cytokines (which might drive infection to a lethal course), they also induce deleterious effects upon thymus, particularly by triggering apoptosis of developing thymocytes [19]. Accordingly, adrenalectomy plus inhibition of GR by the RU-486 compound significantly prevented *T. cruzi*-induced cortical thymocyte depletion [1]. Nevertheless whether or not this is only a systemic endocrine effect or also comprises the paracrine role of intrathymically produced GCs, has not been determined.

Few data are known concerning the effects of PRL during *T. cruzi* infection. Yet, it has recently been showed that PRL supplementation in infected rats is associated with an improvement of the immune response [20]. However, a possible role of PRL (either via endocrine and/or paracrine pathways) in preventing thymic atrophy and the exit of potentially autoreactive T cells remains elusive.

Considering the immunomodulatory role of PRL upon the thymus and the effects caused by GCs, we investigated herein the role of PRL during thymic atrophy and whether intrathymic cross-talk between PRL/GC-mediated circuitries might influence the outcome of the *T. cruzi*-induced thymus atrophy. Here we showed that *T. cruzi* infection subverts the host's endocrine system inducing an abnormally high response of GCs in detriment of PRL signaling to immature CD4^+^CD8^+^ thymocytes, consequently leading to a thymic atrophy outcome. Accordingly, both thymocyte apoptosis and the abnormal appearance of CD4^+^CD8^+^ cells in peripheral lymphoid organs could be significantly prevented in animals treated with drugs that stimulate PRL synthesis.

## Results

### The onset of thymic atrophy is associated to an imbalance of GR and PRLR gene expression

Several research groups have demonstrated that acute *T. cruzi* infection in mice courses with a progressive thymic atrophy caused mainly by the depletion of immature CD4^+^CD8^+^ thymocytes [1], [17], [21]. Previously we reported that the onset of CD4^+^CD8^+^ cell loss occurs after 8 days post-infection (dpi), and is characterized by the increase in the percentage of apoptosis [17]. After 15 dpi, the thymus was highly atrophic, with a reduction of 80% in the numbers of CD4^+^CD8^+^ thymocytes.

**Figure 1:**
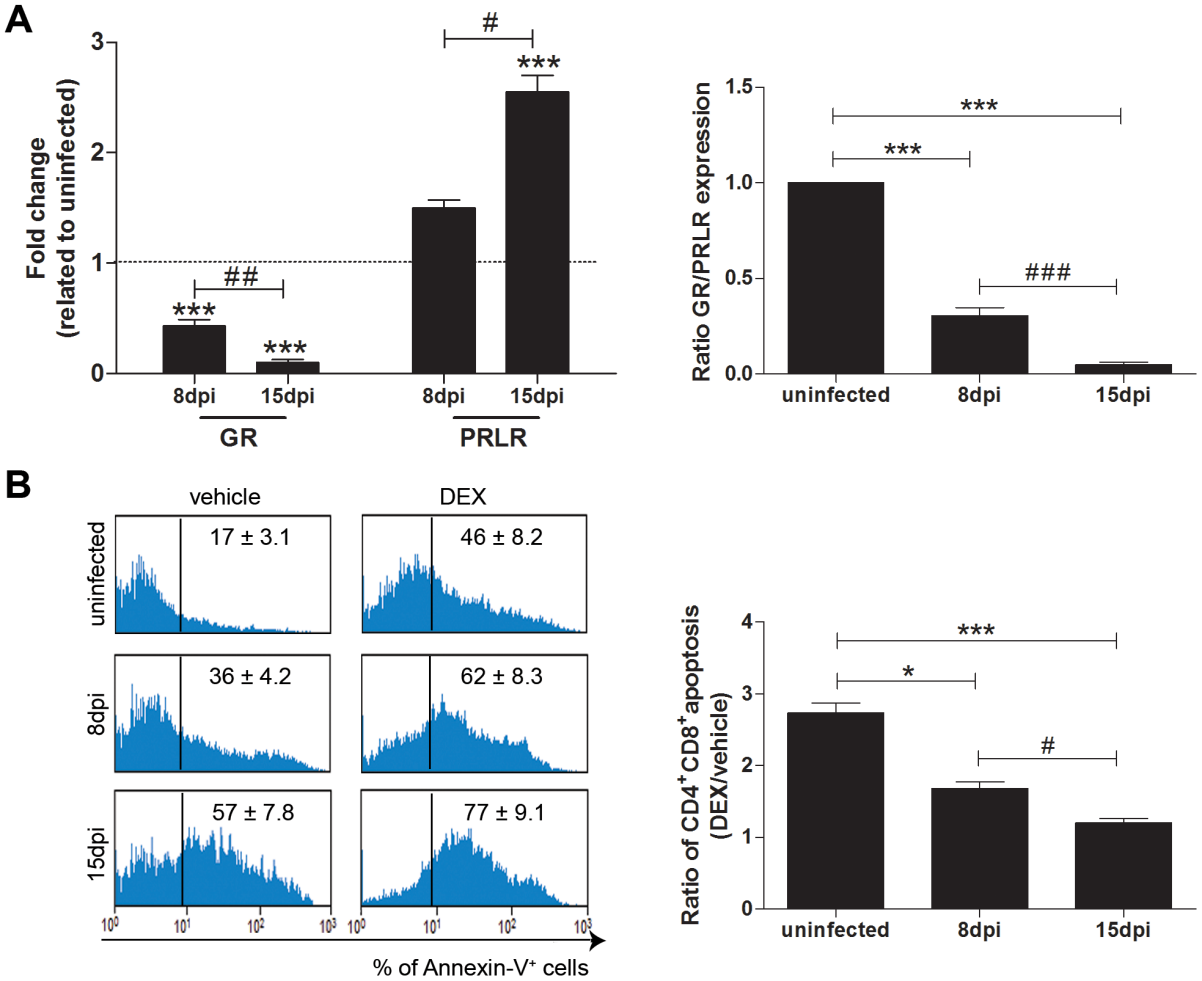
Trypanosoma cruzi induces changes in gene expression of glucocorticoid and prolactin receptors in CD4^+^CD8^+^ cells and alters their sensitivity to GC-induced apoptosis. Panel **A** (left) shows gene expression levels for the receptors for glucocorticoids (GR) and prolactin (PRLR) in CD4^+^CD8^+^ thymocytes, as ascertained by real time quantitative PCR. There is a progressive decrease in GR expression levels during T. cruzi infection, which were paralleled by an increase in PRLR gene expression. The values were normalized to the endogenous reference transcript RPL-13. Values are expressed as fold changes related to uninfected animals and represented as (2**^−ΔΔct^**). Panel **A** (right) represents the ratio of GR/PRLR in CD4^+^CD8^+^ thymocytes during infection. These data are representative of three independent experiments using three mice per group in each experiment. Statistically significant differences (p<0.05) between uninfected versus infected (*) or between 8 and 15 dpi (**#**) mice. Panel **B** (left) depicts progressive increase in apoptosis (revealed by the relative numbers of Annexin V^+^CD4^+^CD8^+^ cells). We can see that in cells treated with vehicle alone or after inbubation with dexamethasone (DEX) there is an increase in the numbers of apoptotic thymocytes. However, if we evaluate the ratio of vehicle-treated over DEX-treated cells (panel **B** right) the sensitivity to DEX-induced apoptosis on CD4^+^CD8^+^ thymocytes is significantly decreased as disease progresses. Thymyses were removed from uninfected, 8 and 15 dpi mice and homogenized. One million cells/animal were incubated with DEX (10^−9^ M) or RPMI (vehicle) during 8 hours under 37°C at 5% CO_2_ atmosphere. Thymocytes were then washed and incubated with anti-CD4/APC, anti-CD8/PercP or Annexin-V/FITC, for the cytofluorometric characterization of apoptosis within each thymocyte subset. These data are representative of two independent experiments using five mice per group in each experiment. Statistically significant differences (p<0.05) between uninfected versus infected (*) or between 8 and 15 dpi (**#**) mice. *p<0.05; **p<0.01, ***p<0.001.

Based on these data we analyzed the expression of the genes coding for GRα and long form of PRLR in CD4^+^CD8^+^ thymocytes from infected mice. We found that these cells progressively reduced GR gene expression during infection, presenting a six-fold decrease after 15 dpi. At the same time, the expression of the PRLR gene increased continuously in this same subset (Fig. 1A, left panel). As result, CD4^+^CD8^+^ cells exhibit a progressive diminution of GR/PRLR expression ratio during infection (Fig. 1A, right panel). The decrease in GR and increase in PRLR gene expression seems to render these remaining cells less sensitive to GC effects. Accordingly, CD4^+^CD8^+^ thymocytes, freshly isolated from infected mice, progressively exhibited a lower apoptosis ratio after being challenged with dexamethasone *in vitro*, compared to the apoptosis triggered when the vehicle alone was applied (Table S1, Fig. 1B left and right panels). By contrast, when these cells were incubated with prolactin, no changes were detected in the apoptosis ratio (data not shown).

### CD4^+^CD8^+^ thymocyte depletion during acute *T. cruzi* infection is associated with systemic and intrathymic PRL-GC hormonal imbalances

As stated above, PRL and GCs are stress-related hormones, while PRL seems to counteract the GCs-induced thymocyte apoptosis [12], [13]. As previously demonstrated, during *T. cruzi* infection PRL levels progressively decreased concomitant to a GC rise in the sera of infected mice (Fig. S1). This hormonal imbalance clearly paralleled the progression of CD4^+^CD8^+^ cortical thymocytes depletion [17].

**Figure 2:**
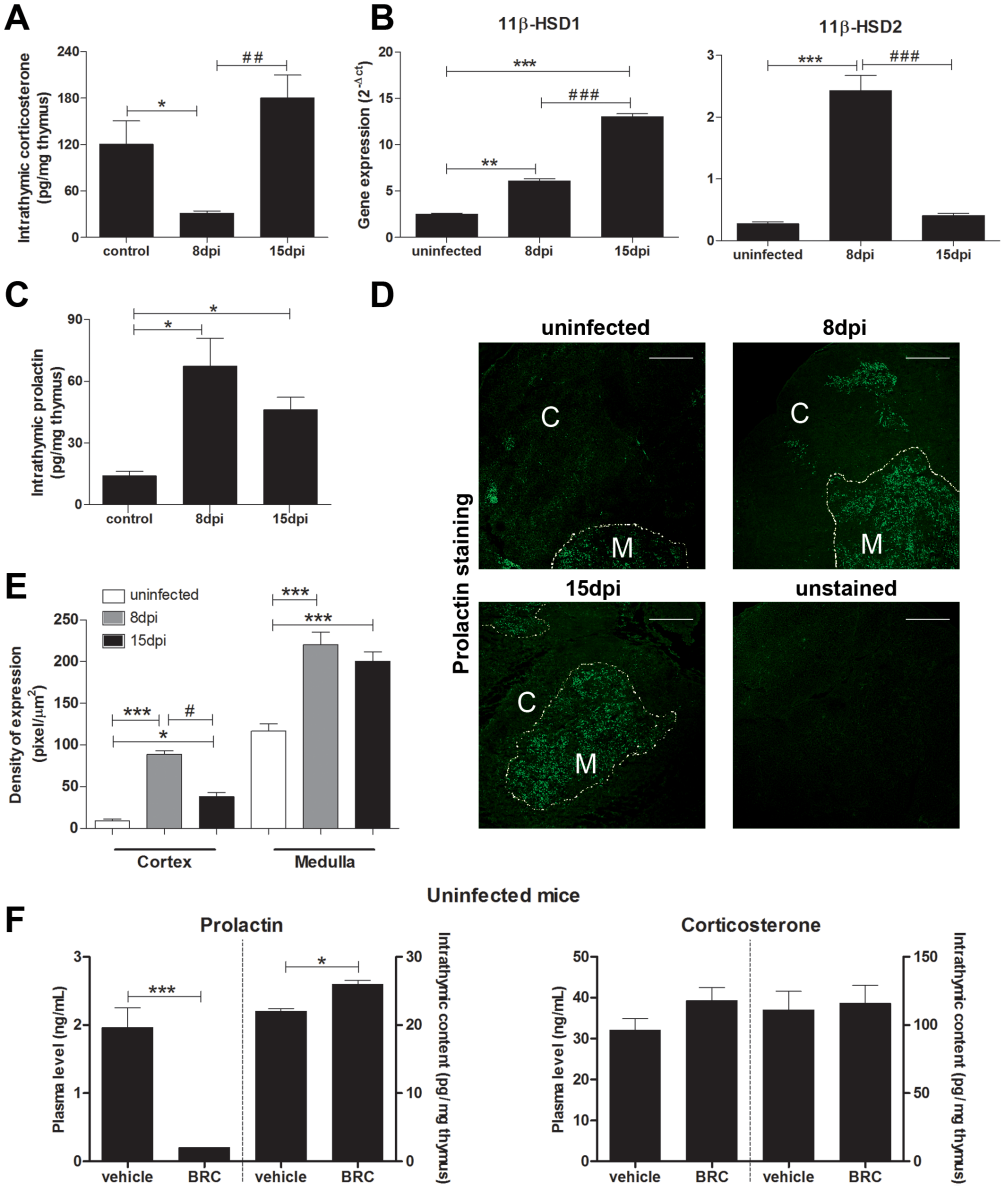
The intrathymic production of glucocorticoids and prolactin is differentially modulated by *T. cruzi* infection. Panel **A** reveals the transient decrease of GC production whereas panel **B** shows the differential gene expression levels of the enzymes 11β-HSD1 and 11β-HSD2. In parallel with the transient decline in GC production, there is a transient increase in prolactin contents as shown in panel **C.** Intrathymic *in situ* prolactin labeling is seen in panel **D**, through representative confocal microscopy images of thymuses from uninfected and infected mice. In this panel, thymuses were stained with the anti-PRL antibody, whereas the lower right box corresponds to the negative control for the immunostaining in which the anti-PRL was replaced by an unrelated goat IgG. The quantification of fluorescence intensity, separately done in the cortex and medulla of thymic lobules, revealed that the rise in PRL contents occured in both regions, as seen in panel **E.** As expected, bromocriptine (BRC), as an inhibitor of prolactin secretion by the anterior pituitary gland, promotes a shutdown of systemic prolactin levels (compared to the treatment with the vehicle alone), as measured in the blood (panel **F** left). However, injection of this compound into normal mice does not induce a decrease in the intrathymic PRL contents, as seen in panel **F**, when comparing BRC- versus vehicle-treated animals. Indeed, a slight (but significant) increase of PRL in the thymus could be detected in BRC-treated animals. Both intrathymic and systemic corticosterone levels remained unchanged in these animals (panel F, **right**). The intrathymic levels of corticosterone were determined by radioimmunoassay, whereas prolactin contents were measured by ELISA in thymus lysates from normal and infected (8 and 15 dpi) mice. In both cases, results were expressed as pg/mg thymus. The transcript levels of 11β-HSD1 and 11β-HSD2 genes were ascertained by quantitative real time PCR. The results represent the amount of transcripts, in relation to the housekeeping gene RPL-13 as 2^−Δct^. As for the confocal microscopy, the original magnification was ×200, with the scale bars = 20 µm. Graphs in panel **E** correspond to quantification of PRL contents, defined by immunofluorescence in 5 microscopic fields of each thymus from uninfected (n = 3) or infected animals (n = 3), with data being represented as pixels/µm^2^. Male mice were daily given s.c. BRC (100 µg/animal) during three days, when they were sacrificed and their thymuses and plasma obtained. Intrathymic and plasma PRL contents were measured by ELISA, with data being expressed as ng/mL in sera, and as pg/mg in the thymus. Statistically significant differences (p<0.05) between uninfected versus infected (*) or between 8 and 15 dpi (**#**) mice. *p<0.05; **p<0.01, ***p<0.001.

In order to better understand the stress-related hormonal circuits in the context of disruption of thymus homeostasis due to *T. cruzi* infection, we studied the intrathymic expression of both hormones. Infected thymuses exhibited a decrease in the local production of corticosterone after 8 dpi, which was reestablished to uninfected levels after 15 dpi (Fig. 2A). This intrathymic GC fluctuation was related with changes in the 11β-HSD2/11β-HSD1 balance. Compared to controls, the highest levels of 11β-HSD2 gene expression were observed after 8 dpi, while for 11β-HSD1 was observed after 15 dpi. Therefore, increased 11β-HSD2 gene expression at 8 dpi supposed an increase of the active form of the enzyme and could explain the diminution in the thymic contents of corticosterone at the same day, whereas the increased expression of 11β-HSD1 after 15 dpi. overcomes these effects increasing corticosterone levels. (Fig. 2B).

The intrathymic PRL production was also altered during *T. cruzi* infection. PRL was augmented in thymuses from 8 dpi mice (Fig. 2C). Immunofluorescence analyses indicate that medullary thymic epithelial cells are the main intrathymic source of PRL during acute infection (Fig. 2D). Morphometric evaluation showed that PRL was augmented in the thymic medulla in both 8 and 15 dpi thymuses, and a significant increase in the cortex was observed after 8 dpi (Fig. 2E), with a trend to diminish after 15 dpi.

The opposing systemic *versus* thymic modulation of PRL levels during *T. cruzi* infection suggests that each circuitry acts independent to each other, in response to the parasite infection. To investigate this issue, thymuses and blood were collected simultaneously from infected mice treated with bromocriptine (BRC), a potent D2 agonist that inhibits the release of pituitary PRL, or vehicle applied as negative control. As expected, BRC generated a drastic decrease of systemic PRL levels in uninfected mouse (Fig. 2F). Nevertheless, this treatment increased the intrathymic contents of the hormone. Both systemic and intrathymic corticosterone levels remained unchanged in BRC-treated mice (Fig. 2F).

### Systemic PRL impairment in *T. cruzi* infected animals causes an increase of GC levels, which impact upon the thymic atrophy

**Figure 3:**
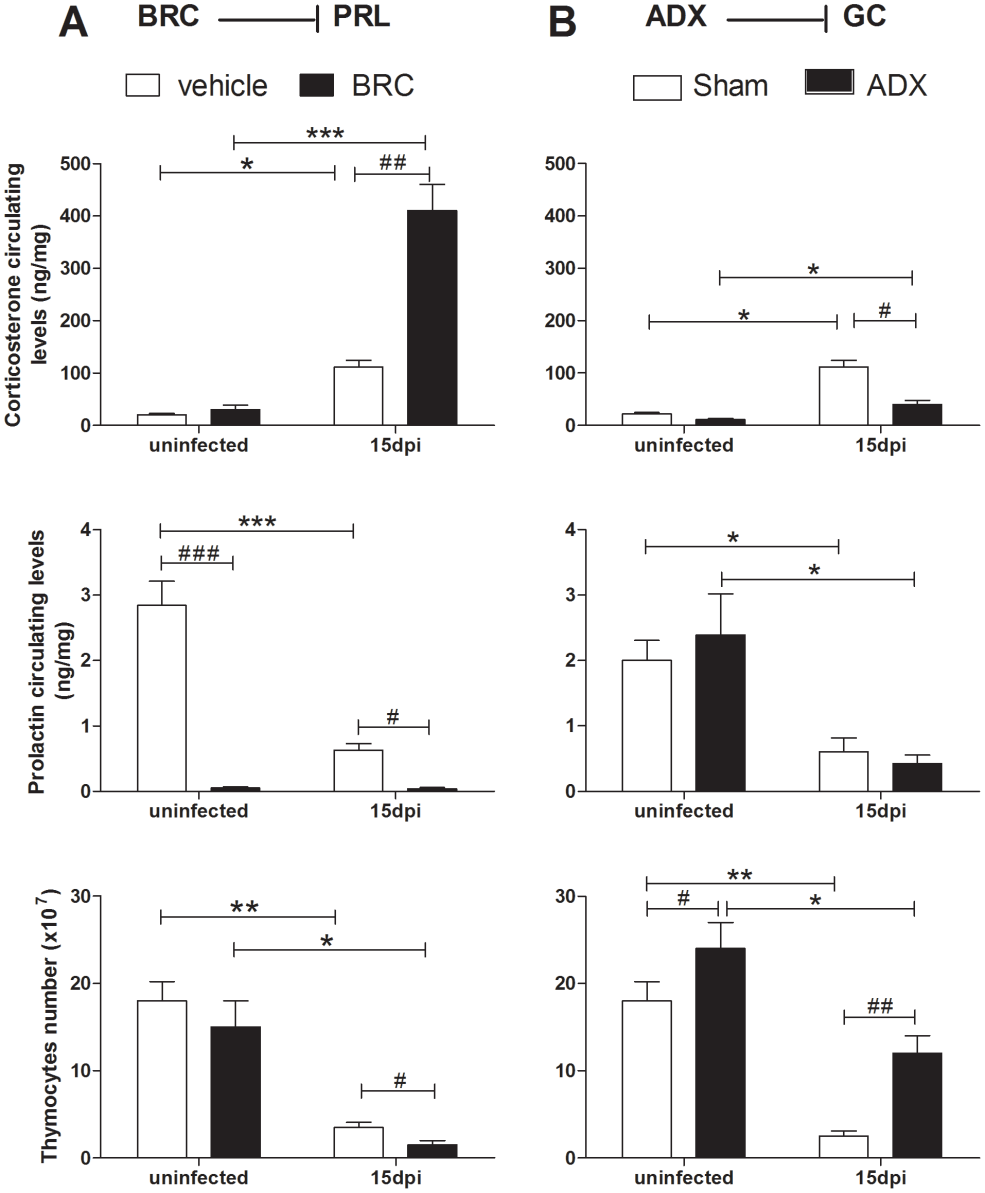
CD4^+^CD8^+^ depletion in *T. cruzi* infected animals is related to the increased systemic GC levels, subsequent to decrease in serum PRL. In panel **A**, the effects of the PRL synthesis inhibitor, bromocriptine (BRC) is shown, whereas the effects of adrenalectomy (thus abrogating adrenal-derived GCs) are seen in panel **B**. BRC-induced shutdown of pituitary PRL production increases serum GC levels, but adrenalectomy does not promote a reciprocal rise in PRL. Moreover, BRC decreases thymocyte numbers in infected animals, whereas ADX promote a significant increase in the total numbers of these cells. Infected and uninfected animals were daily given BRC (10 mg/kg/100 µl s.c.) or vehicle alone for 15 days, when they were killed under non-stressor conditions. Mice were adrenalectomized one week prior to infection, and sham surgery was performed in control animals. Sera were used for quantifying PRL (by ELISA) and corticosterone (by radioimmunoassay). Simultaneously, thymuses were removed and total thymocyte numbers determined. Data are representative of two independent experiments using five to eight mice per group in each experiment. Statistically significant differences (p<0.05) between uninfected versus infected (*) or between 8 and 15 dpi (**#**) mice. *p<0.05; **p<0.01, ***p<0.001.

We have previously identified that GCs are potent stimulators of immature thymocyte loss during *T. cruzi* infection. Nevertheless, nothing was known concerning the role of PRL upon the systemic increase of GCs during infection, nor the possible impact upon thymus atrophy. In order to clarify this issue, mice were treated daily with BRC, from the moment of infection until 15 dpi, when they were sacrificed and their blood and thymus analyzed. Infected animals treated with BRC presented an increase in systemic GC levels of approximately fourfold, and this was associated with decreased thymocyte numbers, when compared to the mice that only received PBS (Fig. 3A), as well as an increased parasitemia [parasites/mL, median (rank), n = 5mice/group; 15 dpi.: BRC group: 9000 (8000–12000), PBS group: 6000 (4000–7000), p<0.05]. Interestingly, a significantly enhancement in GC levels was only observed in infected animals treated with BRC, but not in the uninfected counterparts, thus revealing that this phenomenon resulted as a consequence of *T. cruzi* infection. In an attempt to check a possible cross-talk between PRL- and GC-mediated circuits, we analyzed if the decrease of PRL secretion during infection could be related to the GC rise. For this, we adrenalectomized mice (ADX) one week prior to infection, in order to remove GC systemic contents, and then analyzed the PRL levels and thymuses of the animals. Although thymic atrophy was prevented in ADX infected mice and parasitemia diminished [parasites/mL (n = 5mice/day), 15 dpi: ADX group: 2500 (1000–6000), Sham group: 8000 (5000–10000)], p<0.05)], circulating PRL levels remained decreased in both Sham and ADX animals (Fig. 3B).

Thus, it seems that although in infected mice PRL diminution acts as a permissive factor to the increase in GC synthesis, GC are not related, at least directly, to the diminished PRL systemic levels.

### The re-establishment of systemic PRL by metaclopramide limits *T. cruzi*-induced thymic atrophy

**Figure 4:**
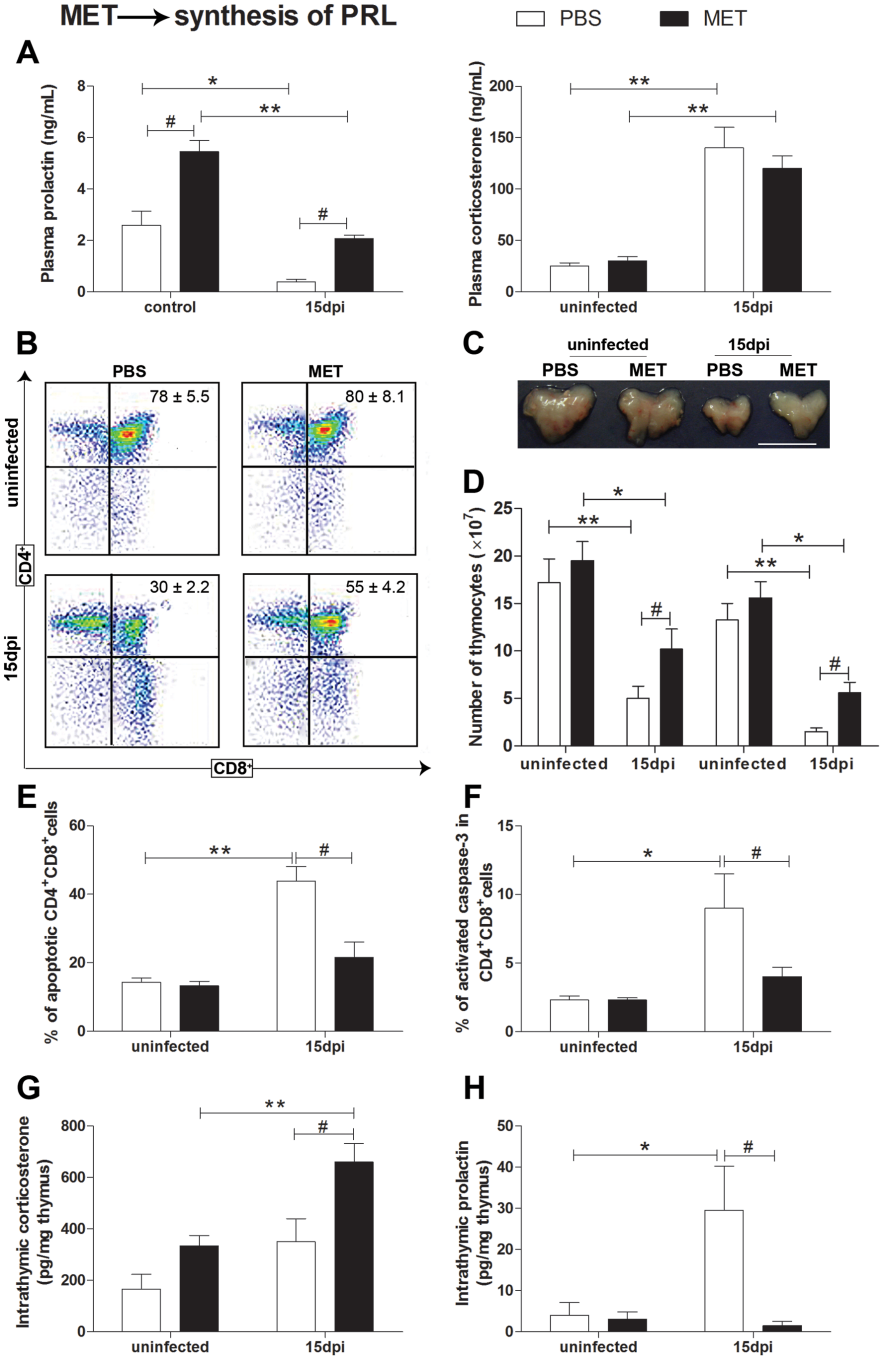
The metoclopramide-induced reestablishment of PRL synthesis in infected mice prevents thymus atrophy. As shown in panel **A**, metoclopramide (MET) does increase PRL serum levels, but without affecting the *T. cruzi*-induced rise in serum corticosterone. Yet, as observed in **B** the CD4^+^CD8^+^ thymocyte subset profiles were partially reestablished in MET-treated infected animals, and so was the size of their thymuses (representative images seen in panel **C**; scale bar = 1 cm). Thymic recovery was further confirmed by the partial yet significant restoration of total thymocyte numbers as well as absolute numbers of CD4^+^CD8^+^ cells (**D**). MET also reduced the percentages of apoptotic CD4^+^CD8^+^ thymocytes, including those exhibiting activated caspase-3 (**E** and **F**, respectively). Intrathymic levels of corticosterone were increased in MET-treated mice whereas PRL decreased, as demonstrated in **G**. Uninfected and infected mice received daily MET (2.5 mg/kg/100 µl, s.c.) from 10 to 14 dpi, or PBS as negative control. On 15 dpi, animals were sacrificed and their thymuses and sera removed for analysis. Cytofluorometric profiles for detection of CD4 and CD8 are shown as dot blots (panel **B**) with inserted numbers corresponding to percentages of CD4^+^CD8^+^ subset as mean ± SE. CD4^+^CD8^+^ apoptotic cells were characterized as AnnexinV-FITC positive staining, whereas the activity of caspase-3 was ascertained as FITC-VAD-FMK positive cells. The values are given as percentage of CD4^+^CD8^+^ cells with active caspase-3. In all cases, data are representative of two independent experiments using five mice per group. Corticosterone and PRL levels were determined, respectively by radioimmunoassay and ELISA. Intrathymic contents are represented as pg/mg thymus and systemic levels as ng/mL. Statistically significant differences (p<0.05) between uninfected versus 15 dpi (**#**) mice. *p<0.05; **p<0.01, ***p<0.001.

Since PRL seems to protect from GC-induced thymic atrophy during acute infection, together with the fact that its depletion worsened the course of disease, we decided to evaluate the effect of PRL re-establishment in *T. cruzi*-infected mice. Animals were daily given metaclopramide (MET) from the day 10 until 14 dpi and then killed. MET administration not only was able to increase PRL systemic levels (Fig. 4A), but also diminished the thymus atrophy (Figs. 4B–C). Interestingly, MET treatment did not induce obvious alterations in GC levels, which remained increased in infected MET-treated mice at the same levels that were seen in the infected PBS-treated mice (Fig. 4A). As expected, MET administration diminished systemic GC/PRL ratio in both uninfected and infected mice (expressed as % of expression change of MET in respect to PBS-treated mice (ie. GC/PRL ratio uninfected = 0.4; Infected-15 dpi = 0.6) Nevertheless, increase in PRL levels did not influence the control of infection [parasites/mL (n = 5mice/day), Infected-15 dpi: MET group: 4500 (3000–7000); PBS group: 5500 (2800–7000), p>0.05] but prevented the loss of CD4^+^CD8^+^ thymocytes in infected mice (Fig. 4D) The degree of apoptosis (Fig. 4E) and also the proportion of cells showing caspase-3 activity were also decreased in these cells, strongly indicating that PRL prevents CD4^+^CD8^+^ apoptosis by inhibiting activation of caspase-3 pathway (Fig. 4F).

Surprisingly, MET administration induced the augment of intrathymic corticosterone and impaired PRL contents in infected mice, which presented a large increase of intrathymic GC/PRL level ratio (expressed as % of expression change of MET in respect to PBS-treated mice (ie. GC/PRL ratio, uninfected = 2.5; 15 dpi = 50).

**Figure 5:**
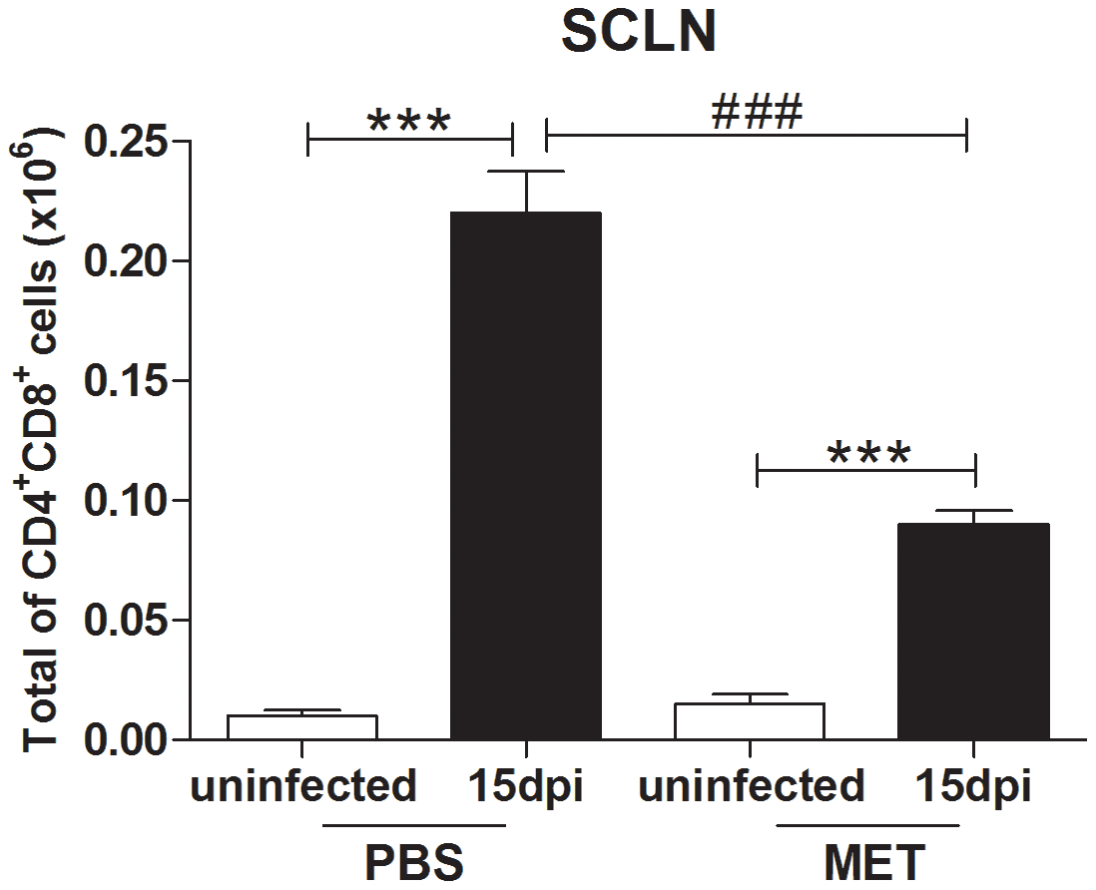
Metaclopramide diminishes the abnormal appearance of CD4^+^CD8^+^ cells in subcutaneous lymph nodes from *T. cruzi* infected mice. The figure shows that in mice treated with the vehicle alone, acute *T. cruzi* infection did induce the abnormal appearance of CD4^+^CD8^+^ cells in subcutaneous lymph nodes (SCLN). Although such cells were also detected after *in vivo* treatment metoclopramide (MET, which promotes an increase in circulating PRL levels) their absolute numbers were significantly lower than those recorded in infected counterparts treated with the vehicle alone. Uninfected and infected mice received daily s.c. MET from 10 to 14 dpi or PBS as vehicle. At 15 dpi they were killed and their SCLN removed, homogenized and counted using Neubauer chamber. One million viable cells were immunostained with anti-CD4, anti-CD8 and anti-TCRβ antibodies, and CD4^+^CD8^+^ T cells were thus characterized. Absolute numbers of these CD4^+^CD8^+^ T cells were expressed as mean ± SE. These data are representative of two independent experiments using three mice per group in each experiment. Statistically significant differences (p<0.05) between uninfected versus infected (*) or between 8 and 15 dpi (**#**) mice. ***p<0.001.

As mentioned above, one of the outcomes of the *T. cruzi*-induced disruption of thymus homeostasis is an abnormal release of immature CD4^+^CD8^+^ to the periphery of the immune system (4, 5). We thus investigated if MET administration in infected animals could also modulate this event. Indeed, we found that MET significantly diminished the absolute numbers of CD4^+^CD8^+^ T cells in subcutaneous lymph nodes of infected mice (Fig. 5). Together, these data demonstrate that decreased PRL directly affects thymocyte viability and leads to the thymus atrophy outcome during acute *T. cruzi* infection.

## Discussion

The host defense mechanisms, during experimental Chagas disease seem to be under thymic control. Congenitally athymic homozygous (nu/nu) mice were shown to be significantly more susceptible to *T. cruzi* infection than their thymus-bearing heterozygous (nu/+) [21]. Moreover, adult thymectomy increased chagasic myocarditis [22].

We have previously shown that thymic atrophy during acute experimental Chagas disease is characterized by a massive loss of CD4^+^CD8^+^ thymocytes, being related to the pro-apoptotic action of increased levels of circulating GCs [1], [19]. Such depletion is associated with a systemic immunoendocrine imbalance, in which GCs and TNF-α are involved [19], [23]. The precise mechanisms underlying this phenomenon are not completely elucidated, but they are likely linked to a particular pathogen-host relationship established during infection. Moreover other hormones could be involved in this altered response. Here we analyzed the involvement of PRL in these abnormalities.

It is well known that PRL and GCs are stress hormones, which systemic levels are increased during adverse *stimuli* [24], [25]. Under these conditions, increased PRL could protect the host from an exacerbated immunosuppressive response mediated by GCs. Nevertheless, during murine *T. cruzi* infection this balance seems to be disrupted, since we observed a progressive decrease of systemic PRL paralleling the enhancement of GCs, as previously demonstrated [17]. This hormone imbalance was already significant at 8 dpi, when we detected the onset of thymus atrophy, which increased along with disease progression. It is known that PRL and GCs participate in many aspects of thymus physiology, presenting opposite activities, which ultimately contribute to the maintenance of the organ homeostasis [7], [14]. CD4^+^CD8^+^ thymocytes from infected mice presented reduction of GR-α transcript simultaneously to an increase in the long form PRLR gene expression. This GR-α is generated through an alternative use of exon 9β, and is highly related to the immunosuppressive properties of GCs [26], [27]. On the other hand, the signaling generated by long form of the PRLR, the main isoform presented in the thymus [28], is strongly associated with biological activities related to the Stat5-P transcriptional activities, which include the prevention of GC-induced apoptosis in T cells [29]. This inverse regulation of GR/PRLR might be a consequence of alteration of GC/PRL ratio. Previous findings have pointed that increased levels of GCs down-modulate GR and up-regulate PRLR [30]–[32]. CD4^+^CD8^+^ thymocytes that remained at 15 dpi presented a lower sensitivity to GCs-induced apoptosis associated both to GR and PRLR modulation, suggesting these receptors may interact influencing CD4^+^CD8^+^ viability. It seems that the changes in GR/PRLR ratio are related to an intrinsic ability of these cells to be protected against the pro-apoptotic action of increased systemic GCs during *T. cruzi* infection.

Although pro-inflammatory cytokines released during infection have been identified as potent stimulators of GC synthesis by the adrenal gland [1], [33], nothing is known on the role of PRL upon the systemic GC rise seen in experimental Chagas disease. The pharmacological blockage of PRL synthesis during the whole course of infection caused an additional increase of circulating corticosterone and a more severe thymus atrophy, when compared to infected animals that received vehicle alone. This data suggest that the systemic PRL production is necessary to control the secretion of GCs and their effects upon the thymus during *T. cruzi* infection. However, we did not detect any alteration of PRL levels in infected mice lacking circulating GCs. Which factor(s) determine the decrease of PRL secretion during infection remain(s) unknown. It is possible that pro-inflammatory cytokines systemically available alter hypothalamic circuits. In keeping with this possibility, both IL-6 and IL-1β have been described to inhibit PRL secretion by the anterior pituitary [34]. MET administration, which inhibits dopaminergic receptors located in the hypothalamus, did recover PRL systemic levels, showing that the control mechanisms of PRL secretion by the pituitary gland are preserved during infection. Thus, although the adenopituitary function seems to be impaired during *T. cruzi* infection [35], the pituitary synthesis of PRL can be restored in infected individuals through the use of PRL secretagogues.

The systemic scenario seems to differ from the one observed intrathymically. Our results reveal that the onset of the thymic atrophy, in the early phase of acute infection (8 dpi), occurs in parallel with the modulation of intrathymic contents of both PRL and GCs. However, contrasting to what was observed in the serum, there was a local PRL increase together with an impairment of corticosterone production, suggesting that the intrathymic synthesis of these hormones does not depend on the systemic circuitry It is known that the availability of corticosterone in thymus is related with a local 11β-HSD2/11β-HSD1 balance. While 11β-HSD2 mainly catalyzes the conversion of biologically active corticosterone in its inactive derivative 11-dehydrocorticosterone, 11β-HSD1 acts in the opposite way, determining the local contents and the availability of GCs. However, the precise mechanisms involved in the intrathymic synthesis of PRL are not elucidated. It seems that extrapituitary cells secrete PRL by pit-1-independent mechanisms [36]. Moreover, systemic and intrathymic regulation events are independent, favoring the idea of separate endocrine niches inside the thymus. The precise involvement of locally produced hormones for thymus atrophy is not clear, but we can conceive that the inverse modulation related to systemic levels that occurs at the initial phase of atrophy corresponds to compensatory intrathymic events so that to restore the organ homeostasis, similar to what has been reported for local production of hormones in different organs [37], [38].

In the last set of experiments, we checked if the reestablishment of PRL systemic levels could prevent *T. cruzi*-induced thymic atrophy. The administration of MET in later acute infection, increased circulating PRL and restored thymus cellularity, diminishing the loss of CD4^+^CD8^+^ by apoptosis, inhibiting the activation of caspase-3. Interestingly, the short period of MET administration prevented thymus atrophy without altering parasitemia or systemic GC levels, thus indicating a direct effect of PRL upon thymocyte survival.

It should be pointed out that we only achieved our results working with a low dose of MET, able to reestablish circulating PRL of infected mice to levels quite similar to uninfected mice. This is in keeping with the data reported by Tomio and co-workers, showing that only low doses of PRL are able to activate PRLR on T cells, whereas higher doses rather suppress this response [39].

Together, our results indicate that PRL counteracts GC effects in situations of increased GCs, by a cross-talk action directly affecting GR signaling in CD4^+^CD8^+^ cells. As previously mentioned, PRL suppresses many GR transcriptional activities by the induction of Stat5-P, a transcriptional factor related with the PRLR/JAK pathway [40]. In this way, it has been described that PRL protects thymocytes from GCs-induced apoptosis both *in vitro* as *in vivo*, by the induction of anti-apoptotic proteins, like bcl-2 and XIAP, which inhibit caspase activation [12], [13].

Importantly, reestablishing systemic PRL through MET stimulation not only prevented thymic atrophy, but also significantly decreased the numbers of CD4^+^CD8^+^ cells in the periphery of the immune system, as ascertained in subcutaneous lymph nodes of infected mice. Since these activated and potentially autoreactive cells are abnormally released from atrophic thymuses [3]–[5], it is conceivable that PRL-mediated thymus protection also influences in the abnormal export of these immature T cells.

**Figure 6:**
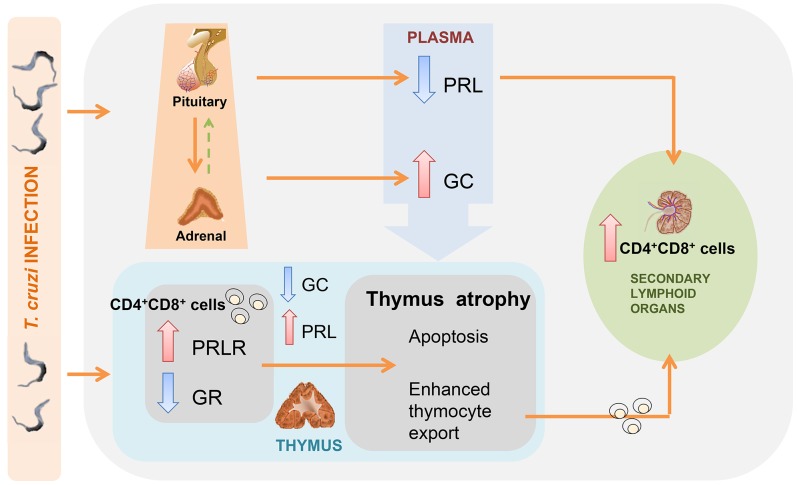
Representative scheme illustrating how *T. cruzi* infection possibly alters the endocrine status resulting in thymus atrophy. Trypanosoma cruzi infection impairs PRL synthesis by the anterior pituitary and increases corticosterone release by the adrenal. The decrease of circulating PRL seems to directly affect corticosterone synthesis, inducing its increase in circulation. This hormonal imbalance affects intrathymic homeostasis, increasing locally produced PRL simultaneously to the decrease of intrathymic GC contents. Simultaneously, GR progressively diminishes at CD4^+^CD8^+^ while PRLR increases. The alteration in the expression levels of both receptors seems to be associated with the increasing sensitivity to apoptosis showed by the CD4^+^CD8^+^ thymocyte subset. Additionally, systemic variations of PRL levels promote the occurrence of potentially autoreactive CD4^+^CD8^+^ cells in periphery, phenomena clearly observed during acute and chronic *T. cruzi* infection.

In conclusion, our findings clearly show that *Trypanosoma cruzi* subverts mouse thymus homeostasis by altering intrathymic and systemic stress-related endocrine circuitries with major consequences upon the normal process of intrathymic T cell development, as schematically illustrated in figure 6. Moreover, exogenously induced enhancement of prolactin secretion partially restores normal thymocyte development, reducing the apoptosis of these cells by inhibiting the pro-apoptotic action of GCs, as well as the numbers of immature CD4^+^CD8^+^ T cells in the periphery of the immune system.

As final consideration, evidence showed here supports the notion that the immune and the neuroendocrine systems act in coordinating a defensive response during infectious processes. Nevertheless, these protective mechanisms may create an adverse state that affects thymic homeostasis. The possible causes for the observed *T. cruzi* infection-associated endocrine disturbances include several and not mutually exclusive possibilities. Immune derived cytokines can directly act at the hypothalamus-pituitary level and/or upon peripheral glands, such as the adrenals. *Moreover, in situ* inflammatory reactions promoted by the parasite or their antigens in the endocrine microenvironment may lead to a transient immuno-endocrine dysfunction.

## Materials and Methods

### Ethics statement

This study was approved by the Animal Research Ethics Committee of the Oswaldo Cruz Foundation, Rio de Janeiro (protocol CEUA-0145-02) and the Bioethics and Biosecurity Committees of Faculty of Medical Sciences from National University of Rosario (Resolution N°3913/2009). All animal experimentation was performed in accordance with the terms of the Argentine and Brazilian guidelines for the animal welfare regulations in accordance with international guidelines.

### Animals, drug treatment and infection

Male BALB/c mice, aging 6–8 weeks, were obtained from the animal facilities of the Oswaldo Cruz Foundation (Rio de Janeiro, Brazil) and the National University of Rosario (Rosario, Brazil). Tulahuén strain was maintained by serial passages in the Vero cell line. Mice were infected subcutaneously with 100 viable trypomastigotes, counted using Neubauer chambers. To monitor the systemic repercussion of the acute disease, parasitemia and analysis of lymphoid organs were recorded following infection, where parasitemia was evaluated using the Brener method, as previously reported [1]. In order to modulate the intrapituitary synthesis of PRL, bromocriptine (BRC) and metoclopramide (MET), D2-dopaminergic agonist and antagonist, respectively, were administrated subcutaneously (s.c.) in uninfected and infected mice. Drugs were obtained from Sigma-Aldrich (St. Louis, EUA). BRC was dissolved in ethanol∶saline 0.9% (2∶8) and daily administered at the concentration of 10 mg/kg/100 µl during the whole infection period. MET was diluted in PBS and administrated from 10 to 14 dpi, at a final concentration of 2.5 mg/kg/100 µl. In each condition, a group of animals was inoculated with the respective vehicle as control.

### Adrenalectomy

Mice were anesthetized with 100 mg/kg ketamine and 2 mg/kg xylazine and bilateral adrenalectomy was performed via a dorsal surgical approach. Two small incisions were made on each side of the back just below the rib cage and the adrenal glands were removed with curved forceps. Sham mice were operated in a similar manner, but without removing the adrenals. The animals were used for further experimentation 1 week after the surgery.

### Determination of systemic levels of corticosterone and prolactin

Mice were housed individually for 1 week before the experiments were started and kept single-caged throughout the experiments in temperature-, humidity-, and light (12 h light: 12 h darkness cycles)-controlled rooms. Samples for hormone determinations were obtained by tail bleeding between 08.00 and 10.00 a.m., after 8 and 15 dpi, as well as from uninfected mice. Blood was centrifuged during 10 min at 1,500 rpm at 25°C and serum was stored frozen at −20°C until analyzed. Corticosterone levels were determined by RIA (MP Biomedicals, New York, USA) as previously described [41], whereas PRL concentration was evaluated by specific ELISA kit, according to the manufacturer's specifications (R&D Systems, Minneapolis, USA).

### Determination of intrathymic hormone contents

Simultaneously to blood collection, thymuses were removed, weighted and homogenized in PBS with protease inhibitors cocktail (Hoffmann-La Roche Ltd, Switzerland). After centrifugation at 12,000 rpm at 4°C, during 10 min, supernatants were kept at −80°C until used. Prolactin and corticosterone levels were determined as described above. Final hormone concentrations were represented by the ratio of hormone concentration in supernatants and thymus weight.

### *In situ* detection of prolactin and GC in the thymus

The pattern of intrathymic PRL localization was ascertained by indirect immunofluorescence assay. Thymuses were removed from uninfected mice and after 8 and 15days of infection, embedded in Tissue-Tek (Miles, Elkhart, USA) and frozen in liquid nitrogen. Five µm-thick cryostat sections were settled on poly-L-lysine (Sigma, Missouri, USA) covered glass slides, acetone fixed and incubated with PBS-BSA 1% to avoid nonspecific binding of fluorochromes. Samples were submitted to specific goat anti-mouse prolactin polyclonal antibody (Santa Cruz Biotechnology, California, USA), 1∶50 for 1 hour at room temperature, washed and submitted to appropriate secondary antibody, 1∶500 Alexa 488-coupled goat anti-rabbit IgG (Molecular Probes, Eugene, USA). Negative control was obtained by substituting primary antibodies by an unrelated goat IgG. Samples were analyzed by confocal microscopy using a LSM 510 Zeiss device (Germany) and the images obtained were subsequently analyzed using the Image J software (Bethesda, Maryland, USA).

### Cytofluorometry and enrichment of CD4^+^CD8^+^ thymocytes

Thymuses were removed, homogenized, washed and resuspended in PBS containing fetal calf serum 5% (Gibco, California, USA). For analysis of thymocyte subsets, cells (1×10^6^ thymocytes/animal) were resuspended in flow buffer and incubated with specific monoclonal antibodies for 30 minutes at 4°C in the dark (anti-CD4/APC, anti-CD8/Percp, BD Pharmingen, San Diego, USA). CD4^+^CD8^+^ lymphocytes in lymph nodes were detected using additional anti-TCRβ/FITC, for the characterization of T cells. Apoptotic cells were identified by means of fluorescein isothiocyanate (FITC)-conjugated Annexin-V (BD Pharmingen, San Diego, USA). Necrotic cells were excluded using propidium iodide (PI). The caspase activity was detected using the caspase-3 detector FITC-VAD-FMK (CaspACE, California, USA). For each acquisition, once the T cell gate was defined, 20,000 events were recorded. Background staining values, obtained with fluorochrome-matched IgG isotype controls, were subtracted to establish specific fluorescence intensity. Fluorescence was measured using an FACS Canto II flow cytometer (BD Biosciences, California, USA) and the percentages of positive cells for each labeling were determined using the FlowJo software (Tree Star, Oregon, USA). For cell sorting, thymocyte subsets were FACS isolated from three pooled thymuses obtained from mice at 8 and 15 dpi, using a MoFlo cell sorter (Beckman Coulter, Indianopolis, EUA). The CD4^+^CD8^+^ subset thus obtained was used for further analysis. All cells were enriched to purity greater than 97%.

### *In vitro* assay for thymocyte-induced apoptosis

Thymuses were removed from uninfected, 8 dpi and 15 dpi mice and homogenized. One million cells/animal were incubated with dexamethasone (10^−8^ M) or RPMI (vehicle) during 8 hours at 37°C, in a CO_2_ incubator. After this period, cells were washed and subjected to the anti-CD4/APC, anti-CD8/Perc or Annexin-V/FITC, for the cytofluorometric characterization of apoptosis within each thymocyte subset. This assay was also performed alternatively incubating thymocytes with prolactin (10^−9^ M). Drugs were obtained from Sigma-Aldrich (St. Louis, EUA) and dissolved in RPMI.

### Gene expression in CD4^+^CD8^+^ thymocytes and total thymuses

RNA was extracted from CD4^+^CD8^+^ enriched cells (10^6^ per group/dpi) using a commercially available kit (RNA easy minikit, Qiagen, Courtaboeuf, France). For total thymuses analysis, RNA was isolated using the guanidine thiocyanate kit (Invitrogen, California, USA) following the manufacturer's instructions. RNA quality and quantity were assessed using an Agilent bioanalyzer (Caliper Technologies Corp., Massachusetts, USA). First strand cDNA synthesis was prepared with 0.5 µg total RNA, random hexamer primer, and SuperscriptIII reverse transcriptase (Invitrogen, California, USA). For qPCR we used approximately 60 ng of cDNA from each sample and SYBR Green Master Mix 2 (Applied Biosystems, California, USA). cDNA was amplified using specific murine primer sequences described in Table 1. All reactions were run on the ABI 7500 Sequence Detection System (Applied Biosystems, California, USA). After 40 cycles of amplification, expression of CYP11A1 (cytochrome P450, family 11, subfamily a, polypeptide 1), STAR (steroidogenic acute regulatory protein), 11β-HSD1 (hydroxysteroid 11-beta dehydrogenase 1), 11β-HSD2 (hydroxysteroid 11-beta dehydrogenase 2), GR-α (Nr3c1, Nuclear receptor subfamily 3, group C, member 1) and long PRLR was assessed by comparing the expression of each to the normalizer RPL-13a (ribosomal protein L13A) using the Ct method as previously described (2^−ΔCt^×1,000) [42], subsequent to the following primer efficiency analysis. Each experiment was run in triplicate with different cDNA preps from the same mice. Genebank assessment number for each of these genes can be seen in Table 2.

**Table 1:** Nucleotide sequences of primers used for the analysis of transcripts and the size of the corresponding amplicons.

Transcript	Oligonucleotide sequence (5′ to 3′)	Product size (bp)
**RPL-13**		180
FW	CCAAGCAGGTACTTCTGGGCCGGAA	
RV	CAGTGCGCCAGAAAATGCGGC	
**GR-α (Nr3c1)**		64
FW	CAAGTGATTGCCGCAGTGAA	
RV	CATCCAGGTGTAAGTTTCTGAATCC	
**long PRLR**		148
FW	ATCATCACAGTAAATGCCACGAAC	
RV	GATGACAGCAGAAGAGAACGGCCAC	
**CYP11A1**		83
FW	GACCTGGAAGGACCATGCA	
RV	TGGGTGTACTCATCAGCTTTATTGA	
**STAR**		69
FW	TCACTTGGCTGCTCAGTATTGAC	
RV	GCGATAGGACCTGGTTGATGA	
**11β-HSD1**		94
FW	TGGTGCTCTTCCTGGCCTACT	
RV	CTGGCCCCAGTGACAATCA	
**11β-HSD2**		79
FW	CCGTGTTCTGGAAATCACCAA	
RV	AATATTGAGGCCAGCGTTGTTAA	

**Table 2:** Symbols, GenBank accession numbers and descriptions of the genes mentioned in the text.

Symbol	GenBank number	Description
**Rpl13a**	NM_009438.5	Ribosomal protein L13A
**Prlr**	NM_011169.5	Prolactin receptor, transcript variant 1
**GR (Nr3c1)**	NM_008173.3	Nuclear receptor subfamily 3, group C, member 1
**Cyp11a1**	NM_019779.3	Cytochrome P450, family 11, subfamily a, polypeptide 1
**Cyp11b1**	NM_001033229.3	Cytochrome P450, family 11, subfamily b, polypeptide 1
**Star**	NM_011485.4	Steroidogenic acute regulatory protein
**Hsd11b1**	NM_008288.2	Hydroxysteroid 11-beta dehydrogenase 1
**Hsd11b2**	NM_008289.2	Hydroxysteroid 11-beta dehydrogenase 2

### Statistical analyses

Differences in quantitative measurements were assessed by the Kruskall-Wallis non-parametric analysis of variance and Mann-Whitney U test. Correlations were evaluated by Spaerman test (non-parametric). Results were expressed as mean ± standard error (SE) unless otherwise indicated. The GraphPad Instat 4.0 software (GraphPad, California, USA) was applied for statistical analyses, and differences were considered significant when p value was <0.05.

## Supporting Information

Figure S1 ***Trypanosoma cruzi* infection results in a hormonal imbalance of prolactin and corticosterone systemic levels.** The graphics clearly show that circulating corticosterone levels progressively increase along with the infection; the opposite occurring in respect to PRL levels in the blood. Sera were obtained from normal and infected (8 dpi and 15 dpi) and kept at −70°C until the analysis. Corticosterone levels were determined by radioimmunoassay and prolactin by ELISA, with the results being expressed as ng/mL. Statistically significant differences (p<0.05) between uninfected versus infected (*) or between 8 and 15 dpi (**#**) mice. **p<0.01, ***p<0.001.
(TIF)

Table S1 **Dexamethasone-induced apoptosis of CD4^+^CD8^+^ thymocytes along with** ***T. cruzi*** **acute infection.** Thymuses were removed from uninfected (control), 8 dpi and 15 dpi mice and homogenized. 10^6^ cells/animal were incubated with DEX or RPMI (vehicle) during 8 hours under 37°C at CO_2_ incubator. After this period cells were washed and incubated with anti-CD4/APC, anti-CD8/Perc or Annexin-V/FITC, for the characterization of apoptosis inside each thymocyte subset. Values represent the ratio of CD4^+^CD8^+^ apoptotic cells.
(DOCX)
