# Supplementary figures and images for: Trypanosoma cruzi Disrupts Thymic Homeostasis by Altering Intrathymic and Systemic Stress-Related Endocrine Circuitries
Source: PLoS Negl Trop Dis. 2013 Nov 14;7(11):e2470. doi: 10.1371/journal.pntd.0002470 (PMC3852165; doi:10.1371/journal.pntd.0002470)

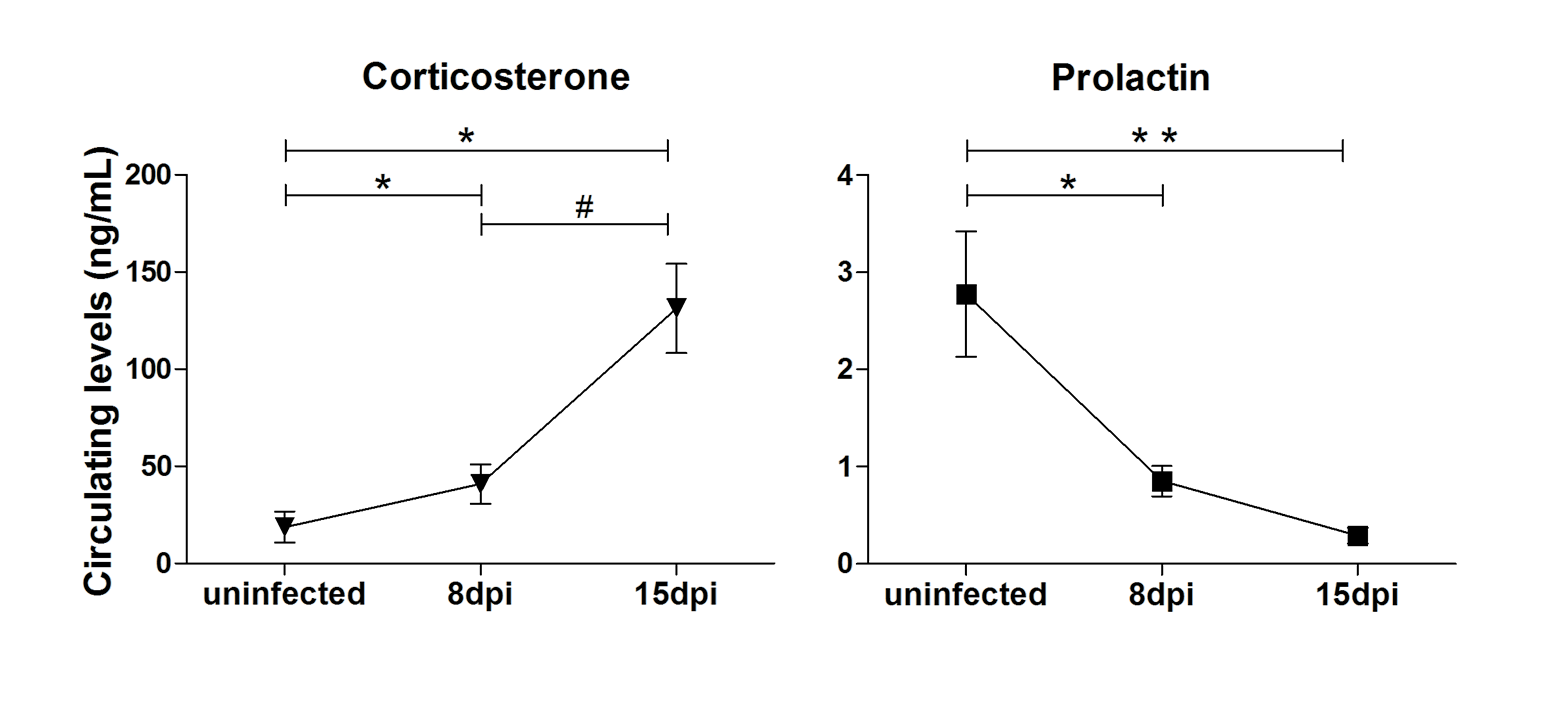

Supplement: Figure S1 — Trypanosoma cruzi infection results in a hormonal imbalance of prolactin and corticosterone systemic levels. The graphics clearly show that circulating corticosterone levels progressively increase along with the infection; the opposite occurring in respect to PRL levels in the blood. Sera were obtained from normal and infected (8 dpi and 15 dpi) and kept at −70°C until the analysis. Corticosterone levels were determined by radioimmunoassay and prolactin by ELISA, with the results being expressed as ng/mL. Statistically significant differences (p<0.05) between uninfected versus infected (*) or between 8 and 15 dpi (#) mice. **p<0.01, ***p<0.001. (TIF) [file pntd.0002470.s001.tif]
