# Supplementary material for: Trypanosoma cruzi Disrupts Thymic Homeostasis by Altering Intrathymic and Systemic Stress-Related Endocrine Circuitries
Source: PLoS Negl Trop Dis. 2013 Nov 14;7(11):e2470. doi: 10.1371/journal.pntd.0002470 (PMC3852165; doi:10.1371/journal.pntd.0002470)
Supplement: Table S1 — Dexamethasone-induced apoptosis of CD4+CD8+ thymocytes along with T. cruzi acute infection. Thymuses were removed from uninfected (control), 8 dpi and 15 dpi mice and homogenized. 106 cells/animal were incubated with DEX or RPMI (vehicle) during 8 hours under 37°C at CO2 incubator. After this period cells were washed and incubated with anti-CD4/APC, anti-CD8/Perc or Annexin-V/FITC, for the characterization of apoptosis inside each thymocyte subset. Values represent the ratio of CD4+CD8+ apoptotic cells. (DOCX) [file pntd.0002470.s002.docx]

**Table S1. Dexamethasone-induced apoptosis of CD4^+^CD8^+^** **thymocytes along with *T. cruzi* acute infection**

|  | **vehicle** | **DEX (10^-9^M)** |
| --- | --- | --- |
| **control** |  |  |
| 1 | 17 | 46 |
| 2 | 20 | 52 |
| 3 | 15 | 40 |
| 4 | 22 | 54 |
| 5 | 16 | 45 |
| **8dpi** |  |  |
| 1 | 36 | 62 |
| 2 | 44 | 65 |
| 3 | 40 | 58 |
| 4 | 35 | 50 |
| 5 | 41 | 55 |
| **15dpi** |  |  |
| 1 | 57 | 78 |
| 2 | 72 | 88 |
| 3 | 60 | 71 |
| 4 | 55 | 70 |
| 5 | 53 | 71 |

Thymuses were removed from uninfected (control), 8dpi and 15dpi mice and homogenized. 10^6^ cells/animal were incubated with DEX or RPMI (vehicle) during 8 hours under 37ºC at CO_2_ incubator. After this period cells were washed and incubated with anti-CD4/APC, anti-CD8/Perc or Annexin-V/FITC, for the characterization of apoptosis inside each thymocyte subset. Values represent the ratio of CD4^+^CD8^+^ apoptotic cells.
